# Housing and health for people from refugee and asylum-seeking backgrounds: findings from an Australian qualitative longitudinal study

**DOI:** 10.1186/s12889-024-18616-5

**Published:** 2024-04-24

**Authors:** Anna Ziersch, Moira Walsh, Clemence Due

**Affiliations:** 1 Flinders University, College of Medicine and Public Health, Flinders Health and Medical Research Institute, Adelaide, Australia; 2 School of Psychology; University of Adelaide, Adelaide, Australia

**Keywords:** Refugee, Asylum seeker, Housing, Neighbourhood, Social determinant, Integration, Longitudinal, Qualitative, Ontological security/insecurity

## Abstract

**Background:**

For people from asylum-seeking and refugee backgrounds, housing and the re-establishment of home are key social determinants of health. Research highlights the inequities faced by asylum seekers and refugees in the housing markets of high-income resettlement countries, resulting in their overrepresentation in precarious housing. There is also emerging evidence of the relationship between housing and health for this population relating to lack of affordability, insecurity of tenure, and poor suitability (physical and social). The mechanisms by which housing impacts health for this group within these housing contexts, is however, understudied - especially overtime. This qualitative longitudinal study aimed to address this gap.

**Methods:**

Semi-structured interviews were conducted with 25 people from asylum-seeking and refugee backgrounds in South Australia, recruited through a community survey. Thematic analysis of interview data across three time points over three years identified four material and psychosocial mechanisms through which housing contributed to health outcomes via psychological and physical stressors - physical environment; stability; safety; and social connections, support and services. The study also identified additional health promoting resources, particularly elements of ontological security. The dynamics of these indirect and direct mechanisms were further illuminated by considering the impact of international, national and local contexts and a range of intersecting social factors including gender, country/culture of origin, family circumstances, immigration status, language skills, income, and health status.

**Conclusions:**

Rebuilding a sense of home and ontological security is a key resettlement priority and crucial for wellbeing. More comprehensive strategies to facilitate this for refugees and asylum seekers are required.

## Introduction

There are currently, 108 million people displaced worldwide as refugees and asylum seekers [1]. Involuntary displacement, experiences of persecution, violence, and loss of family, community resources, signals disconnection from place that cannot be easily rebuilt, especially in resettlement countries [2, 3]. Recent studies have also shown much higher prevalence rates of anxiety, depression, and post-traumatic stress disorder (PTSD) in refugees that are resettling in high-income countries compared to non-refugee populations [4, 5]. For those who are forced to seek refuge, accessing appropriate housing, re-establishing a connection to place and creating a sense of home provide a pathway to rebuilding ontological security [6–8]. Conversely, difficulties accessing suitable housing present barriers to this - contributing to and compounding post migration stressors [9, 10].

Housing is established as a key social determinants of health (SDH), and a recent review highlights housing context/location (neighbourhood), physical elements, affordability, housing markets and housing policies as key areas through which housing impacts health [11]. Emerging research shows that this association may be especially critical for refugees and asylum seekers [12–14]. This article builds on this work through the analysis of interviews undertaken over three-years with refugees and asylum seekers in South Australia. Specifically, this paper seeks to examine what the mechanisms are that drive the relationship between housing and health over time for people from asylum- seeking and refugee backgrounds? To answer this question, we employed a SDH framing [15], drawing on socio-ecological understandings of health [16] and elements of integration [17]. This framing considers the multilevel, cumulative, and reciprocal relationship between housing and health and the wider contexts (social, political, material, and economic) that influence day-to-day lives and shape health outcomes [18].

### Terminology

#### Refugee and asylum seeker

The terms ‘refugee’ and ‘asylum seeker’ are used in this paper to refer to people who meet the criteria for refugee status as defined by the United Nations High Commissioner for Refugees [19], and those still waiting for their claims to be assessed, respectively. The term ‘refugee’ is used in this paper to also cover asylum seekers unless immigration determination status is pertinent. However, we acknowledge the limitations associated with both these terms in describing only one aspect of identity.

#### Health and wellbeing

Our use of the term ‘health’ aligns with the World Health Organisation’s definition as “a state of complete physical, mental and social wellbeing, and not merely the absence of infirmity [20].

## Background

### Housing and health

The relationship between housing and health is complex [21–23], and may also be bi-directional with factors such as poor health/disability, education, race and ethnicity, gender and access to services/resources, influencing one’s capacity to secure appropriate housing as well as the health impacts of housing experiences [24–26].

At a more specific level, tangible factors like inadequate shelter, overcrowding, cold and damp, and toxins have a range of negative health consequences [15, 27]. Housing has also been linked to health in terms of psychosocial elements such as privacy, agency/control, empowerment/autonomy, and a sense of being at home. In turn, these factors all contribute to ontological security [6, 7] – a concept defined by Giddens [28] as a sense of identity and constancy in relation to self, as well as social and physical environments. When this sense of constancy is deeply disrupted through fractures in an individual’s life, such as through forced displacement, this can bring about ontological insecurity, “which is both a disruption of the cognitively ordered world of self and other, and the management of individual wants” [29].

According to Dupuis and Thorns [30], the markers of ontological security in one’s home are: (1) material and social constancy; (2) a place where daily routines can be performed; (3) a sense of control and freedom from public surveillance; and (4) a place of security where identities can be constructed. Precarious housing – defined by Mallett and colleagues [24] as comprising two or more of the following elements: (un)suitability, (un)affordability and (in)security of tenure – has been shown to inhibit ontological security vis-a-vis the associated lack of agency and control over housing and neighbourhood, with negative impacts on health [6, 29, 31–33].

### Housing and health for refugees

Key links between housing and health for refugees have been established in prior research (see [14] for a review). Physical health effects, for example, have been linked to poor housing conditions (cold, damp), size and layout leading to overcrowding and lack of space, and instability contributing to challenges managing health needs. Mental health outcomes, the focus of most studies in the review, were linked to many of the issues detailed above such as housing condition, security of tenure, mobility, and overcrowding as well as safety, social connections, and experiences of discrimination. While negative mental health effects are associated with precarious housing for migrants more generally, and the general population, the risk of poor mental health to refugees is significantly increased given the higher rates of mental ill health experienced by this group [13, 34, 35]. Housing precarity may also be compounded by precarities related to the refugee journey, such as those associated with employment, access to healthcare and education, legal status, and limited social connections and support [36]. Moreover, in resettlement, refugees and asylum seekers, particularly those who are ‘visibly different’, are more likely than other migrant groups to experience precarious housing [14].

As above, forced migration and heightened levels of threat to personal safety, as well as ongoing uncertainty, constitute major disruptions to one’s life, contributing to an affective state of ontological insecurity. Rebuilding a sense of ontological security is therefore of particular importance for refugees [37]. Challenges in post migration and resettlement contexts, such as prolonged detention, prolonged family separation, limited access to supports and services, discrimination, insecure residency, and limited access to education and employment have been shown to having a compounding impact on mental health and ontological insecurity [38–40].

A range of studies from Australia and other high-income countries have provided insights into the housing experiences of refugees and other migrants [41–47]. These studies highlight problems associated with limited affordable housing stock, experiences of housing discrimination, and risks of homelessness, as well as the importance of social connection to positive housing pathways. Two longitudinal studies have identified that refugees experience the least improvement in their housing circumstances over time [48, 49]. To our knowledge, there is only one longitudinal study on the housing experiences of refugees that focuses on health [13]. Martino and colleagues examined longitudinal quantitative data from Australia across a five-year period from two longitudinal surveys – one focused on the resettlement experiences of refugees and the other on the household, income, and labour statistics of the whole population. They found negative mental health effects that could be attributed to precarious housing for both groups, but this effect was more pronounced for refugees.

### SDH, socio-ecological and integration framing

We situate this longitudinal exploration of housing and health for refugees within a SDH framing [15], which draws on socio-ecological understandings of health [16] and elements of integration [17]. SDH are the ‘conditions in which people are born, grow, work, live, and age...[which] are shaped by the distribution of money, power and resources at global, national and local levels’ [50]. SDH influence health at a range of levels - individual factors (e.g. age, sex), individual lifestyle factors (e.g. smoking, exercise), social and community networks, living and working conditions (e.g. housing, neighbourhood, education, work environment) and general socioeconomic, cultural and environmental conditions [51]. In terms of refugees, these conditions contribute to resettlement and integration experiences, as well as health outcomes.

Integration is a contested concept but is understood here as a two-way process of adaptation that occurs between incoming and receiving communities [17]. Ager and Strang’s [17] influential framework includes ten indicators of integration across four domains. In the ‘Means and Markers’ domain, housing, along with education, employment, and health, are viewed as markers of, and means to, successful integration. The ‘Social Connection’ domain includes social bonds, bridges, and linkages, the ‘Facilitators’ comprises language and cultural knowledge, and safety and stability, and ‘Foundation’ covers rights and citizenship. This model and Dahlgren and Whitehead’s model of the SDH overlay significantly, particularly in relation to the shared markers of SDH and inclusion of health [52] and the potential ways that these features can mutually reinforce one another for refugees [47]. Importantly, a greater focus on the social context of receiving communities and the interrelatedness of the different levels has been called for [53, 54] and to account for intersectional forms of oppression [55] and various aspects of identity and personal histories in the integration process.

As such, we look at health and housing, alongside SDH and integration, in a social-ecological context where it is possible to examine the social policies and processes that influence the housing experiences of refugees and associated impacts on health.

## Materials and methods

The findings in this paper form part of a larger study of the relationship between housing, social inclusion and health for asylum seekers and refugees in Australia, which involved surveying of over 400 people who arrived in Australia as asylum seekers or refugees and interviewing 50 people who indicated in the survey that they were interested in participating further [56]. For this paper we report on 25 participants who took part in at least two rounds of interviews. First wave interviews were conducted March-November 2016. Second round interviews were conducted with 25 participants in May 2018-February 2019, an average of 22.85 months later. Nineteen people then took part in a third-round interview (conducted January-July 2020), an average of 21.26 months after second round interviews.

In Australia at the time of the interviews around 30,000 people were on temporary refugee visas (TVs) (those who arrived by boat after 2012 without a valid visa and claimed asylum were not eligible for a permanent refugee visa (PV) even if their claim was upheld) or awaiting their refugee claims to be processed and were on bridging visas (BVs) [57]. These TVs generally entitled holders to fewer supports. For new arrivals on PVs, housing support was offered in the first 6 months (less than a month for asylum seekers) after which it was expected that they would source independent housing. This was overwhelmingly in the private rental market given extensive waiting lists for social housing.

Table 1 shows participant characteristics. At the time of the second-round interview, seven asylum seekers had had their claims for refugee status accepted and granted a temporary refugee visa, four remained on bridging visas awaiting a decision from immigration, and one remained on a temporary refugee visa. One citizen (at the time of the first round) took part in a second and third-round interview, and one permanent resident (PR) became a citizen between the first and second-round interviews. The remaining 11 second-round participants were PRs. 11 of the 12 TV holders took part in a third-round interview, with two asylum seekers being granted temporary refugee visas just prior. Three third-round participants became citizens between the second and third rounds, and three remained PRs, with plans to apply for citizenship when eligible. In this paper, we use pseudonyms to describe participants and include their gender, visa status and region of origin for any direct quotes we use.

**Table 1:** Participants

	Round 1	Round 2	Round 3
	n=50	n=25	n=19
**Gender**	Women n=22	Women n=8	Women n=6
	Men n=28	Men n=17	Men n=13
**Immigration status**	Permanent visa n=28	Citizen n=2	Citizen n=5
	Temporary visa n=22	Permanent visa n=11	Permanent visa n=3
		Temporary visa n=12	Temporary visa n=11
**Region of Origin**	Middle East n=31	Middle East n=19	Middle East n=14
	Africa n=9	Africa n=4	Africa n=3
	Southeast Asia n=9	Southeast Asia n=2	Southeast Asia n=2
**Time in Australia**	6 months – 7 years	2.5 years – 8 years	4 years – 10 years
	(mean = 5 years)	(mean = 6 years)	(mean = 6.7 years)

### Procedure

The study received ethics approval from the Flinders University (then) Social and Behavioural Research Ethics committee, and the researchers adhered to a range of ethical considerations crucial to working with refugees and asylum seekers. These included paying attention to issues of coercion, power imbalances between the researchers and participants, and ensuring confidentiality and anonymity [58, 59]. A Community Advisory Group made up of refugees and asylum seekers, as well as a broader Reference Group of service providers and other stakeholders helped to guide all aspects of the study. In particular, the interviewing researchers were provided with guidance from experienced service providers in trauma informed and culturally appropriate approaches to engaging with refugees and asylum seekers from diverse backgrounds. This included taking time to connect with potential participants and earn their trust, meeting in a place that was most comfortable for participants, seeking ongoing consent during interviews, and referring participants to services if they required assistance. Over the course of the second and third round interviews, one participant was connected with a counselling service for her son who was described as experiencing depression.

Interview participants were initially recruited through the broader survey [46, 56]. Participants for second (and then third round) interviews were recruited from the original interview pool and the interviewing researcher contacted no more than three times via telephone call or text to seek people’s approval to meet for an interview. Interpreters were offered and used by the same five participants in each round of interviews. In each case, the participants were happy with a professional interpreter.

In interviews, questions focused on participants’ housing experiences and broader resettlement and self-reported impacts on health, with round two and three interviews focusing on changes since last interview. First round interviews lasted between 16 and 70 minutes (mean = 32.23). Second round interviews lasted between 25 and 79 minutes (mean = 44.13). Third round interviews lasted between 22 and 93 minutes (mean = 51.11). Three non-migrant women conducted the first-round interviews. Another non-migrant woman conducted the second and third round interviews. All interviewers had extensive training and experience in conducting interviews with people from refugee backgrounds in relation to health.

### Data analysis

In our analysis we were guided by a critical realist approach [60], which provided the philosophical underpinnings for examining institutional and structural factors to consider in relation to health promoting housing, frequently extending beyond the influence of individual agency. Specifically we used a framework thematic analysis approach informed by Ritchie and Spencer (1994), and Lewis [61, 62]. This involved: familiarisation with the data, where transcripts from the whole data set (across the time points) were read by multiple team members; developing a coding framework and then indexing using the NVivo Version 12 qualitative software database (QSR International, Melbourne, Australia). The coding structure was adapted and refined during team meetings and as the coding took place to allow for outliers in the data. The charting phase involved the research team developing thematic matrices where participants were charted against the emergent themes across the three time points. These matrices were developed so that the team could look across all stages of data collection to “capture an essence of the journey travelled” over time by exploring how changes happened, what these changes looked like, changes to participants’ trajectories, and participants’ narratives of the impacts of their housing experiences over time on their wellbeing in the connect of their refugee journey [62]. All the data were then summarised by interview round and combined so that common and divergent themes across the whole data set could be identified. In the final mapping and interpretation phase, the health-related housing experiences of the participants over two and three waves were outlined, differences between groups and contexts were identified, and explanations for these developed. This final phase was undertaken during regular team meetings and at other times by individual team members in order to capture the ‘devil in the detail’ [63].

### Findings

First round interviews (reported in more detail [46]) identified that housing had an impact on health - particularly mental health - through issues of unaffordability, unsuitability (including physical elements and the social environment where housing is located), and insecurity of tenure including difficulties securing housing. Importantly, in terms of participants’ self-reported baseline health status, many were living with mental and physical health challenges such as chronic pain, sleep disturbances, symptoms associated with anxiety, stress, and post-traumatic stress disorder (PTSD), and persistent negative emotional states that they attributed to pre and post migration factors including housing, as well as immigration status limbo, prolonged family separation, and barriers to employment, which collectively contributed to a sense of ontological insecurity.

**Fig. 1:**
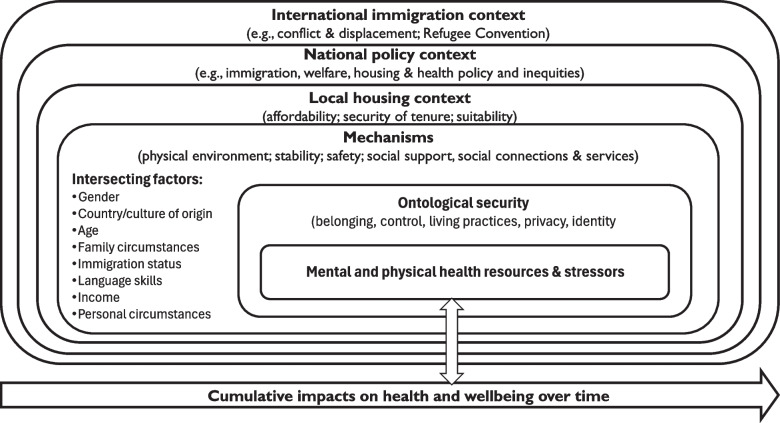
Mechanisms linking housing and health for refugees

Building on the findings from this paper on first round interviews, here we focus on the mechanisms that drive the relationship between housing and health over time. The mechanisms, which are often overlapping, are: physical environment; stability; safety; and social connections, support and services. We highlight the psychosocial elements contributing to ontological security as a pathway to health: belonging, control, living practices, privacy, and identity, and the direct health impacts via mental and physical stressors and access to health promoting resources (see Fig. 1). The findings also reflect a nested socio-ecological approach which considers the relationship between housing and health over time. This includes individual and community level moderating factors (gender, country/culture, family circumstances, immigration status, language skills, income, personal philosophy, and health status) through to features of the local housing context (affordability, security of tenure, suitability) which constitute degrees of precarity, through to the national policy context (e.g., immigration, welfare, housing and health policy and inequities) and international immigration context (e.g., conflict and displacement, the Refugee Convention).

### Physical environment

The analysis revealed that problems with the physical environment (including cold and damp, overcrowding and housing located near noisy traffic) were a key mechanism through which challenges with affordability and suitability impacted health. This included through increased physical and mental stressors, and barriers to rebuilding ontological security. For example, Nahal and her family had moved between the first and second round of interviews to a more affordable house; however, the new house was in poor condition, and she was worried about the impact of mould on her family’s health, especially her infant nephew:

*... because of this mould the baby gets sick [...] so he been in hospital for five days, the baby, and my mum gets very bad pain in her legs and knee and her back because if there is mould *(Nahal, woman, PR, Central and South Asia, 2^nd^ interview).

The family’s inability to afford housing that was in better condition and the housing management’s inadequacy in addressing the issue contributed to Nahal and her family’s distress and worry: *“we’ve been there like two years now [...] just struggling, struggling, struggling.”* This was compounded by their distress regarding the wellbeing and safety of family who remained in the family’s country of origin, and while her nephew was a welcome blessing *– “the baby make our life more happy [...] When I come I see his smile, my care just go away”*, their sense of ontological insecurity was high given their worry for his health. Nahal was too busy with study and caring responsibilities to take part in a third interview; however, she indicated in correspondence with the interviewing researcher, that the family had moved to a larger and much cleaner house, which had eliminated her concerns regarding the mould and improved her health.

Financial precarity and a sense of ongoing hopelessness with the physical environment of their housing was mainly evident in the narratives of TV holders subjected to ongoing visa insecurity. Adeeb, a single young man from Central and South Asia, had moved several times since arriving in Australia as an asylum seeker. At the time of the second interview, Adeeb said he had overstayed his welcome with family, was in significant debt, remained in limbo over his immigration status and did not have work rights. As a result, he had moved into a crowded share house with other single asylum seeker men that was “really old [with] lot of holes around it, [so] the wind come in.” He goes on:

*It’s noisy. [I] can’t sleep at night, you can hear a lot of noise is – it is near the road. With the traffic the house is shaking at the night* (Adeeb, man, TV, Central and South Asia 2^nd^ interview).

Because of the overcrowded physical environment, Adeeb slept on the couch to avoid sharing a room with three other men:

*I just sleep in the lounge room because if you sleep in the [bed]room you need to share with someone else; I don’t like share [...] It’s difficult to stay at that home but because of the rent, it’s a bit cheaper, that’s why I need to stay until I fix all of the money I borrow from my friends, from family. Until six year I live in Australia without [support] because I wasn’t allowed to work*.

By the third interview, Adeeb now had work rights and was working but remained in significant debt and uncertainty about his immigration status. After seven years, and in a housing situation that did not provide privacy or safety, Adeeb continued in a state of ontological insecurity and had all by given up:*I’m just lying [to] myself. I will get a visa. I will buy a house, whatever. That’s all a lie. I’m just kidding myself. *Adeeb’s feelings of hopelessness were further impacted by his confusion and sense of injustice that many members of his own community who arrived around the same time as him had been accepted as refugees and were able to be reunited with their families:

*It’s really difficult for anyone but for me as well because the people, we come by the same boat, they all have visa, they’re applying for citizen, they bring their family here and [they visit] their family but for me still nothing. It’s very difficult. It’s a hard situation for me.*

Lana, a TV holder from the Middle East and mother of two teenaged children discussed affordability in each of her interviews, particularly finding a suitable house in the area that they wanted their children to go to school. She and her husband were unable to find work and had therefore made a trade-off between their desired neighbourhood and a small and old unit on a busy road. By the time of her third interview, after six and a half years in the unit Lana was in despair, describing the impacts on her children’s sleep and mental state:

*Now my son has depression, my daughter has depression. I asked them what happened to you, why they don’t like to talk, they’re always inside their room. They said oh mum, we don’t like this house, always shaking, a lot of noise, we cannot sleep well during the night.* (Lana, woman, TV, Middle East, 3^rd^ interview).

For TV holders, the mental health impacts of living in an unsuitable physical environment were exacerbated by the participants’ lack of control in improving their situation and ongoing ontological insecurity, which were linked to financial precarity, and lack of financial support associated with their TVs, and compounded by stressors associated with forced migration, namely loss of status, language and employment difficulties, and family separation. For Lana and her husband, migrating to another country was not a choice: *“we must do it. Because our country is not safe [...] people they live there already die.”* Both in their late 40s, learning English and finding work that suited their age, skills and qualifications seemed impossible, adding to the family’s sense of ontological insecurity: *“my husband has 30 years’ experience [in the electric] field, but he can’t work because he doesn’t have certificate [and] labour work for him is really hard because he has age”.*

Conversely, improving the physical environment of one’s housing produced resources important to health, particularly through elements of ontological security such as a sense of control and independence. For example, Griva, a PR from Southeast Asia described the impact of her family living in cramped conditions for two years. She had moved into a larger house with four bedrooms just prior to being interviewed for the second time during which time she said:

*The main thing that we have is our own privacy because honestly in that house I nearly got depression [...] sometimes you need time for yourself [...] I didn’t have that earlier. We have our own privacy [...] It’s much better. We have our own room and stuff* (Griva, woman, PR, Southeast Asia, 2^nd^ interview).

When interviewed again, 18 months later, the family was in the same house and although Griva described being diagnosed with clinical depression over this period as a consequence of varied resettlement experiences such as adjusting to the Australian education system, loneliness, and concern for her mother’s mental health. She reiterated the positive impact that having a private space of her own had: “*I can just be myself and then have my own space.”*

Georgieta, an Australian citizen from Africa, during her third interview also described the impact of attaining privacy and space: *“My partner found this house [...] It feels good because just me and my partner living here. There’s no other third party, we have our privacy.”* Over the study, Georgieta had gone from emergency youth accommodation due to multiple suicide attempts, to staying temporarily with an acquaintance while waiting to secure supported accommodation for young pregnant women at risk, to another share house, to a unit with her partner. She reported experiencing significant traumatic experiences associated with political unrest in her country of origin prior to coming to Australia, being shunned by her family and community after being sexually assaulted by a respected community member, having her young children removed from her care and experiencing intimidation and racist attacks from neighbours and sexual harassment from a flatmate. For Georgieta, having a place of her own with her partner provided a sense of privacy and independence, important to rebuilding ontological security.

A small number of participants also spoke of taking control of what they could to improve their physical environment and engage in living practices in housing that was unsuitable, in view of limited other options as highlighted above. Kazem, for example, a qualified tradesperson in his country of origin, spoke of making significant improvements to his rundown rental to cultivate a home more in keeping with his family’s needs:

*[Interpreted] He renovated the kitchen himself for the wife because he knows how the wife likes to live in a nice house and with a nice kitchen and all that. He said back in [my country] we had a good life and good house and everything* (Kazem, man, TV, Middle East,^3rd^ interview).

Likewise, Hiranjan spoke of his vegetable garden during the second and third interview. Although he was concerned about the associated cost of water, he “still love[s] to grow vegetables” (man, PR, Southeast Asia). At the time of the third interview, Hiranjan took great pride in showing the interviewing researcher his garden, and particularly the different vegetables native to his country of origin. Cultivating therapeutic spaces through living practices such as gardening, was a strong theme drawn from several of the first-round interviews [33, 46].

### Instability

Over the course of the study, all but one participant (a social housing tenant) remained in private rental housing. Broader housing unaffordability and difficulties securing housing meant that several participants had no choice but to remain in unsuitable housing rather than face the rental housing market. As above with Lana and Adeeb, this included navigating limited income, unaffordable rents in preferred areas (close to schools and safe neighbourhoods) and challenges having rental applications approved often due to their immigration status. For example, several TV holders from Central and South Asia and the Middle East remained in unsuitable housing over the course of the study due to the challenges associated with applying for rentals as non-citizens. All had unsuccessfully applied for multiple other rental properties, As Bijan shared:

*We tried to move but we weren’t successful [...] It’s hard to find because most of the house here is old house and if you want to find [a real estate agent or owner] you can connect with it’s hard, you know. They’re asking too much [questions] they not give you [house] easy. You know, it’s hard. Moving is very hard* (Bijan, man, TV Middle East, 2^nd^ interview).

TV holder Kazem who had taken control by improving the physical environment of his house (detailed in the previous theme) was similarly unable to find more suitable accommodation for his family: “*looking into the passports and knowing that you’re from Australian background, that’s one of the things that the landlords and the agents are mostly looking.”*

Several participants spoke of renting housing directly from owners, and in some cases owners from the same country of origin. For example, Lachina, a TV holder from the Middle East had prioritised some sense of stability over suitability by renting from a landlord from the same country for seven years. At the second interview she said:

*...my house is old and it was very damaged and it was so dirty, not clean. That house had one point, my owner was [from same country]... and I could very well connect to him* (Lachina, woman, TV, Middle East, 2^nd^ interview).

By the third interview, the family was looking for a new rental because their landlord planned a redevelopment; however, their applications had been rejected several times including from a house that remained advertised. Lachina believed this was because: *“when we sent our documents for [the real estate agent], oh, he finds that we are travel documents in passport”.* The cumulative health effects that Lachina was experiencing were significant:

*I am in stressed, affected in my sleeping, I can’t have a good sleep and more time my allergy comes up and I scratchy skin, and I can’t deep breath, easy breath I can’t. All time I use, I smoke cigarette, it’s very bad for me.*

In terms of stable housing, participants indicated that a key goal was home ownership. While this is the goal of many people in Australia, home ownership and the security it presents was particularly important for this group. For example, Hiranjan from Southeast Asia was focused on his goal of buying a house soon along with other education, employment and travel goals aimed at improving his circumstances. He had experienced unsuitable housing at the time of the second interview while pursuing his education. However, when interviewed for the third time he was working fulltime, aiming to undertake further tertiary study and was resolute in the goal of stable housing through purchasing a home: *“I’m working now, I feel that I can buy home”* (Hiranjan, man, PR, Southeast Asia).

For those on TVs, home ownership was a symbol of a more certain future. For example, by the time of the third interview, Naweed had moved with his wife between rentals and accommodation with friends and then family. He described his wish to have a home of his own:

*It’s my dream to – yeah, freedom with my wife and like our own property [...] I am tenant; I’m not the owner. It’s a big change for me is one day that I can get a good property for myself. I relax when I get home for myself, which is difficult [...] When I’m thinking after 10 years, we can’t do anything, it will affect me and I’m thinking that I do not belong to this country* (Naweed, man, TV, Central and South Asia 3^rd^ interview).

Here Naweed’s TV status is a clear barrier to rebuilding elements of ontological security (belonging), where he has waited in limbo for over a decade for resolution of his asylum claim including long periods without work rights – where ‘we can’t do anything’. Other TV holders highlighted financial limitations associated with their visa as a barrier to home ownership including several who had unsuccessfully applied for a bank loan to purchase a home due to lending conditions associated with their TVs (larger deposit required compared to citizens and much higher interest rates), which made purchasing a house impossible. For example, Shabir, from the Middle East, had remained in unsuitable but affordable housing with the aim to relocate to his own home once he was able to afford to buy. Shabir arrived in Australia with no English and had worked hard to develop his language skills and to earn enough money for a deposit. He had moved from one form of shared accommodation to another much less suitable shared accommodation (more crowded and poorer condition) because the rent was cheaper and described the mental stressors he was experiencing largely due to his unsuccessful and hard-fought attempts to progress and build a sense of belonging and a future in Australia through buying his own house:

*At this age, I’m now 23 or 24 but I look like 30, 35 now because I’m thinking ‘oh family; okay, this much [money] for family. I’ve got this much now’ I need to pursue my future and all these things. It’s not that easy; it’s really difficult [I’m] a lot depressed [...] There is not any option for me. The only option is to [keep going]* (Shabir, man, TV, Middle East, 2^nd^ interview).

In contrast to the ongoing constraints experienced by TV holders, some participants who were PRs discussed improvements in housing situations over time due to factors such as secure immigration status, education, and English language skills and employment providing a sense of stability, control, belonging and optimism for the future. At the second interview Pazir, a PR from Central and South Asia, said he and his family had traded between elements of precarious housing by moving from a costly rental in an area populated with members of their cultural community where they were very happy, to more affordable supported accommodation which did not meet their other needs (space, condition, location). Five years on, as new citizens, Pazir described ‘paying his dues’ in his previous housing and finally relocating with his family to a house that was significantly more suitable:

*...it’s a very good house, very, very good, and very – and a good price, as well [...] like the way my mum, she always wanted. So, we spent really hard time on that house [previous house] That time was like really tough, everything went slowly, slowly. The only thing you have to be [is] patient, I think. If you’re thinking everything will click [into place], or everything there will be magic, that’s not going to happen – with anyone* (Pazir, man, citizen, Central and South Asia, 3^rd^ interview).

Having a sense of control over housing together with other elements of resettlement (employment and education) and stability in immigration status led to key elements of ontological security and positive health outcomes in resettlement. Notably, by virtue of the restrictions associated with their visas, TV holders experienced more barriers and frequently described the mental stressors of having limited control over their access to stable housing and associated sense of belonging. This state of ontological insecurity was compounded by family separation and remittance responsibilities and experiencing ongoing limbo.

### Safety

The extent to which people felt safe in their neighbourhoods was identified as a key mechanism by which elements of housing (affordability and suitability in terms of location) influenced health and access to rebuilding ontological security. As identified in the first round interviews [46], threats to safety and proximity to social disorder (violence, drug and alcohol abuse, anti-social behaviour) were widely reported and prompted people to relocate to safer suburbs. For example, Edris a PR from the Middle East, detailed several burglaries in his first government-provided house, and the effect that feeling unsafe had on his mental health. At the second interview, Edris described having to leave his country of origin due to “problems with the government” that meant it was unsafe for him to stay. Feeling safe in his new environment was therefore critical to his chances of rebuilding a sense of ontological security. Edris and his partner had relocated at this time to an apartment block with a range of security features. Of the move, he said:

*Positive is it’s a good area, as I said, a safe area. We don’t need to be worried [that we will be robbed] when we go out for one day or two days with friends which has happened in our first home two times [...] now we just go out and relax. Don’t need to think about the house.*

Likewise, George a PR from Africa, had struggled through a period of living in a government-provided housing complex soon after arriving in Australia with his mother and siblings, where he and his family were in proximity to a range of anti-social behaviours that led him to feel unsafe. At the time of the second interview, George had a fulltime job in his area of expertise in another State of Australia and was living in a share house where a significant proportion of his neighbours were from his cultural community. He described his housing as “more peaceful.” At the third interview, he was still in the same house and expanded on his sense of feeling safe and at “home”, despite being separated from family still in his country of origin:

*I do feel like home. Whenever you are safe you can call it home, however sometimes you may not be able to see your family members or your people who talk in your language. Sometimes it can be hard but as long as I’m safe, as long as I have very good friends here, so I’m very happy to call it home.*

Edris and George described income from employment, subsidised accommodation and cultural community connections that contributed to their safer living environments. As an avenue to rebuilding ontological security, relocating to a safer neighbourhood contributed to feeling more at home.

Other participants who remained in unsuitable housing in safe neighbourhoods, also acknowledged the importance of safety particularly in the context of their experiences as refugees. For example, Nahal a PR from Central and South Asia and Lana a TV holder from the Middle East were in unsuitable housing that contributed to physical and mental stressors for themselves and their families. However, at their second interviews, they still described the safety they felt in their neighbourhoods as critical to their health:

*I do feel safe. Oh my God, I feel really safe here. If I compare with my country, 90 percent I’m safe here. In my country 90 percent not, ten percent I’m safe. At home you just stay at home. [Here] I go to the beach by myself I’m not scared I’m happy (Nahal).*

*I am happy because when my children go outside I’m sure they come back but in [home country] it’s not possible, really difficult. You don’t know what happens (Lana).*

Most participants over the course of the study had been able to relocate from neighbourhoods where they felt unsafe, through advances in their circumstance (education and employment) or by making trade-offs in terms of the condition of the actual dwelling as seen in a previous theme. However, a small number of participants described experiencing ongoing threats to their safety, which had consequences for their health. For example, William, a PR (at second interview) from Africa, described *“facing hell with the neighbours now”.* Although William expressed high levels of satisfaction with his housing previously, at the second interview, he had relocated to another flat in the same complex and described being harassed by a neighbour causing great worry for his children who all have medical conditions:

*We are living in [continual] fear [the children] are not safe. If we are to take a decision to go back they are not safe in our country, so you see the situation we find ourselves? [...] why are we treated as if we are shit? What have we done? They are pushing people to the edge. *

During the second interview, William was highly agitated and left abruptly after receiving a phone call from his wife, who was suffering from significant mental health issues related to their experiences with their neighbours. The importance of ongoing safety to rebuilding ontological security and health and wellbeing for people from refugee backgrounds is crucial precisely because of the extreme fear already experienced though forced migration and the significant ongoing concerns for family still in conflict zones.

### Social connections, support and services

Over time, housing affordability and suitability (social and physical in terms of location) was reported to impact health through proximity to social connection, support and services. A significant proportion of participants indicated a cultural imperative to have good connections with close neighbours, primarily to aid the development of social and emotional resources important to health through elements of ontological security – namely belonging and identity. While some were able to develop these relationships over time and enjoy the associated resources, others had less success. For example, Chaghama, a PR from Central and South Asia described moving far from her preferred neighbourhood to a more affordable location. Of her new neighbourhood, Chaghama said:

“*If we live ten years still we don’t know who is our neighbour [...] because we were new. [...] We wanted to talk so when we see this we were shocked ‘oh God, it’s so difficult here because nobody, neighbour, not even asking ‘do you need any help? Do you need food or do you – any problem you can call us, you can get anything you want’. You know, if there is a new neighbour come in our side we go and ask ‘do you have food for yourself now? If you’re tired we can cook for you. If you need water, you need anything, please ask us’. When we came here that was a very difficult one, very difficult.*

During the second interview, Chagma described having high levels of distress in relation to the safety and health of her family in her country of origin, including a son who was very unwell. Chagma described how important proximity to community connections was for emotional support around family separation. While she was able to visit with her cultural community connections from time to time, her new neighbourhood was mainly Anglo-Australian and she was yet to develop English language skills meaning she felt unable to make the sorts of connections that would provide longed for social and emotional resources, and which would enable living practices that support ontological security. With chronic physical and mental health issues, the absence of neighbourhood social supports was challenging “*even if I am dying, even something happening, they’re *[neighbours] *not going to come and check with me.”*

Conversely, others described great success with developing strong social bonds with neighbours from the same and different cultural backgrounds, which yielded a range of social and emotional resources positive for health and wellbeing. Farhad and Hiranjan both PRs and from the Middle East and Southeast Asia respectively, described developing family-like bonds with neighbours from Anglo-Australian backgrounds that facilitated feelings of belonging:

*[My neighbour] told me herself a few months ago – or last year I think – she said ‘I would never think that I will have a refugee friend like you that changed my life forever’. I said ‘my God, thank you so much’. [That] kind of conversation and talking just clear for everyone that we are human like you. We are not from a different planet, you know what I mean? [...] now she calls me ‘son’ which is really important. [O]ur friendship is very close and tight now (Farhad). *

*One of my neighbour is ...like my grandmother, yeah, she’s Aussie and she live alone I meet every day. We [have things] like presents on birthday and I invite her for dinner at my home and she always coming (Hiranjan).*

Farhad was also linked in with other friends through his neighbour and took pleasure in noting the large network he now enjoyed where support was reciprocal: “*whenever they need help I’m there to help them and whenever I need help they are there to help.” * While Farhad did not take part in a third interview, Hiranjan did and indicated that he had reluctantly moved to a different neighbourhood as the owner of his rental house was selling. He was hopeful of connecting with his new neighbours and reflected on how critical the relationship with his elderly Australian neighbour was, including the practical resources he gained from the connection, particularly English language skills and cultural knowledge:

*When I was there, we used to pretty much every time we meet each other and having that conversation as well like every maybe I can say half an hour every day. So doing that one, see my English is improving as well, so yeah, it’s really great to chat with her because it’s good to have that all the experience and then like different cultures. Yeah, she shared me a lot* (Hiranjan, man, PR, Southeast Asia 3^rd^ interview).

Developing good relationships with neighbours was also noted as a potential antidote to experiences of discrimination and harassment in one’s neighbourhood, enabling ontological security to be rebuilt. For example, in contrast to the second interview, described in the previous theme, William’s third interview described a close relationship that had developed between a neighbour and his family. The neighbour had recognised the strain that the family was experiencing and had offered to drive the children and William’s wife to where they needed to be if William was at work:

*He’s a very good man, he’s a very generous man. This is one of the reasons that is really, really stopping at the moment to look beyond to seek other accommodation. Because my wife is not driving [...] we found this man, really, really, really helpful* (William, man, PR, Africa, 3^rd^ interview).

In addition, a small number of participants noted changes in proximity to services and other places important to health. Pazir a PR from the Central and South Asia had previously described his time in unsuitable housing and during the third interview expressed great relief that he and his family were able to secure a rental that met all their needs. These needs included proximity to medical care for his mother, who had poor mental and physical health, as well as cultural shops, and the Mosque:

*So comfortable and so amazing, and especially, it’s very near to all the - if you look to the city, it’s very easy. Like, my mum, her doctor’s sitting in [general practice], like 10 - 15 minutes to drive from here, and especially like all the halal butchers, and all the halal shops, Afghani shops, are there, and if you look, there is a mosque only six minutes’ drive from here [...] What else you want?* (Pazir, man, citizen, Central and South Asia 3^rd^ interview).

At the time, Pazir had just become an Australian citizen and during the interview was in cultural dress having attended the citizenship ceremony. His new and more suitable housing was described in the context of this big change in his life, which was a key pathway to rebuilding ontological security and reflective of the layers of influence on health indicated in Fig. 1: *“I’m so satisfied, I’m so lucky, and now, finally, too, from today on, I’m a full Australian.”*

### Discussion

This paper drew on SDH, integration, and socioecological framings to examine the mechanisms by which housing experiences affect health over time for refugees and asylum seekers. We considered the refugee resettlement journey and multilevel, cumulative, and reciprocal relationships between housing and health and the wider contexts (social, political, material, and economic) that influence day-to-day lives and shape health outcomes [18].

The findings add to relatively scarce literature highlighting the way housing acts as a SDH for refugees in high-income resettlement countries, mirroring previous findings in relation to the impact of affordability, security of tenure and suitability in the local housing context on health [14]. As outlined in Fig. 1, the analysis identified several key mechanisms through which housing context (affordability, security of tenure and suitability) affected health negatively over time as mental and physical health stressors and positively through health promoting resources –namely the physical environment of the housing and neighbourhood, stability of housing, sense of safety, and access to social connections, support and services. These findings reflect those of the authors’ original study [46]. Individual elements of these have also been identified previously in the literature, for example in relation to precarious housing [13], overcrowding and lack of privacy [64], and the importance of gardens, physical condition, space, layout and privacy and, in relation to neighbourhood, safety, green spaces and proximity to services [33]. Through this model, however, we seek to identify the complex interplay between these features of housing over time, as well as highlighting the way that psychosocial elements of ontological security such as belonging, control, living practices, privacy and identity were key to these mechanisms [6, 7, 65] and important health resources for refugees and asylum seekers in the context of ontological insecurity that is a by-product of forced displacement [33, 66, 67].

In the accounts of the participants, the cumulative health impacts over time of negative housing trajectories were evident and noted as occurring alongside other overlapping elements of SDH and integration such as challenges with employment, access to education, financial precarity, English language acquisition, migration pathway/temporary visa status, and health status itself. These compounding problems mirror literature pointing to the harm caused by cumulative stressors associated with precarity in multiple aspects of life [13, 68–70]. In contrast, those with existing resources such as English language skills, family networks, and importantly access to supports and services associated with their permanent residency/citizenship status were less susceptible to negative health impacts. The findings from this study highlight the particular precarity experienced by asylum seeker and refugee participants on temporary visas, with most noting the lack of control that they possess as temporary residents of Australia. This lack of (real and perceived) control is not only a psychosocial element of ontological insecurity, but a SDH in its own right [15]. Indeed, ontological insecurity is a key element in asylum seeker and refugee negotiations of agency and control, leading to “the oxymoron of being ‘safe in uncertainty”’ [71].

Intersectional differences across aspects such as gender, country/culture of origin, family circumstances, as well as immigration status, and income were evidenced in the narratives of participants – which reflect broader systems of oppression and privilege such as sexism, racism and classism [72]. These also reflect more broadly national welfare policies and international immigration and the ways that these shape social and health inequities – highlighting the value of a socioecological framing [16]. For example, temporary visa holders reported consistently worse outcomes, indicating how immigration policies around temporary visas and welfare policies around income support, set against a backdrop of increased international forced migration, can help to shape an individuals’ housing trajectory and subsequent health. Aspects of this model reflect the recent model by Swope and Hernandez [23] which seeks to link structures, mechanisms and housing ‘pillars’ to health disparities. We build on this to drill down into aspects of housing that were particularly pertinent to refugees and asylum seekers, who are at greater risk of ill health associated with housing issues [13, 34, 35].

While housing has been identified as an important means and marker of integration [17], the nuanced ways that refugee and asylum seeker housing experiences can (or cannot) support integration, or how the housing market might shape a two-way notion of integration has been less elucidated. We highlight that a hostile private rental market (reflective of broader national and international policy contexts) indicates a receiving community context that is not conducive for successful housing outcomes and integration in this regard [35], and identify the limits of individual agency in navigating this to be able to secure housing that is health promoting.

The housing that resettlement countries provide can support or hinder successful integration and contributes to shaping the health outcomes of resettled refugees. Additionally, consideration of ontological security and an understanding of broader contextual factors is crucial. Many participants continued to experience housing problems, suggesting that housing issues do not necessarily resolve over time and that some refugees and asylum seekers are forced into ‘housing niches’ [73] that are damaging for health. More supportive policies and programs are required to assist refugees and asylum seekers and to transform societies in true two-way integration. Key upstream policies to contribute to this include immigration policies that offer permanency in immigration status and more generous welfare policies to address cost issues, alongside greater regulation of housing markets and incentives for more affordable housing. At a more local level, greater support for refugees and asylum seekers in moving through the private rental market beyond the initial resettlement phase would be helpful, as would efforts to work within local neighbourhoods to foster greater social cohesion and ‘neighbourliness’.

This paper is one of only a small number that have examined the impact of housing and health for refugees and asylum seekers over time. This longitudinal approach assisted in identifying the complex interplay of factors involved and the power (and limits) of individual agency in navigating housing across the resettlement journey. However, limitations include the potential lack of representativeness of the initial sample and that an inability to follow up all of these participants means that those who continued in the study may have had more negative (or positive) trajectories than the initial broader sample.

#### Conclusion

This qualitative longitudinal study identified key pathways between housing and health for asylum seekers and refugees, building on a growing evidence base highlighting housing as a key SDH for general populations and extending this to a resettlement context. It also indicated influences at varying socioecological levels and pointed to key policy levers that must be pulled to improve outcomes for new arrivals. Rebuilding a sense of home is crucial for those experiencing forced relocation, and in the context of two-way integration receiving communities have a crucial role to play in facilitating this process.

## Data Availability

The datasets generated and/or analysed during the current study are not publicly available due to the need to preserve the confidentiality of research participants.
